# The Effect of Sleep Quality and Mental Health on Academic Performance Among the Medical Students of King Abdulaziz University

**DOI:** 10.7759/cureus.44951

**Published:** 2023-09-09

**Authors:** Khaled A Yaghmour, Sara M Alhmyri, Buthaina M Alhmyri, Renad Sharaf, Mashael A Alasmari, Mawaddah M Almilabi

**Affiliations:** 1 Family Medicine, King Abdulaziz University, Jeddah, SAU; 2 Medicine, King Abdulaziz University, Jeddah, SAU

**Keywords:** student health, medical education, grade point average (gpa), pittsburgh sleep quality index (psqi), dass-21

## Abstract

Background: Sleep quality has an important role in brain functioning and development. Affected sleep quality and mental health can negatively affect the academic performance of college students.

Objective: To assess the effect of sleep quality and mental health on the academic performance of medical students at King Abdulaziz University, Jeddah, Saudi Arabia.

Methods: We conducted a cross-sectional study among medical students at King Abdulaziz University. The dependent variable was the current grade point average (GPA). For the independent variables, two validated tools were used in the study: the Pittsburgh Sleep Quality Index (PSQI) for sleep assessment; and the Depression, Anxiety and Stress Scale (DASS-21) for mental health assessment.

Results: A total of 382 responses were analyzed. The majority of students (86.6%) had GPAs greater than 3.75/5, while only 1% of the sample had a GPA lower than 2.75/5. The PSQI showed a median and interquartile range of (9, 6-11). Normal DASS-21 represented the majority as follows: depression at 67%, anxiety at 63.1%, and stress at 82.2%. In the statistical analyses, sleep quality, depression, anxiety, and stress were not statistically significant with the student’s GPA.

Conclusion: Low levels of sleep quality were found among medical students in our study. While sleep quality and mental health status did not show an effect on the GPA of the medical students, lower sleep quality was significantly correlated with increased scores of depression, anxiety, and stress. Our findings mandate interventions directed at improving sleep quality among medical students.

## Introduction

Sleep plays a major role in human life and health, as it is important for brain functions and development. However, the amount of sleep required varies due to normal demographic and biological variations, and not all individuals require the standard eight hours of sleep [1]. Sleep can be affected by various disorders that cause changes in sleeping hours, which may lead to psychological disorders such as depression and negatively affect academic performance [2]. Medical students in particular face an increased risk of sleep disturbances, with studies consistently showing that a significant proportion of medical students (70% to 76%) experience difficulties with their sleep patterns, as measured by the Pittsburgh Sleep Quality Index (PSQI) [3,4]. Furthermore, sleep quality has been found to impact students’ mental health and academic performance [5,6]. Poor sleep quality is commonly reported among college students in the Middle East, with a prevalence of 37.1% in Lebanon and 55.7% in Egypt [7,8].

Interestingly, high achievement has been linked to poor sleep quality in 60% of students, and high achievers are also 42% more likely to experience sleep trouble compared to low achievers. Students who reported sleep troubles also had higher scores on scales for despair, anxiety, and stress [9]. This contradicts the notion that sleep plays a role in facilitating brain function and enhancing concentration [10]. Furthermore, students may be unaware that poor sleep quality can negatively impact their mental health and grades [11].

In Saudi Arabia, little attention is paid to investigating the effect of sleep quality on medical students’ grades. The relationship between sleep and mental health is barely covered in the literature. Therefore, our study aimed to assess the effect of sleep quality and mental health on the academic performance of medical students at a tertiary care university hospital in Jeddah, Saudi Arabia.

## Materials and methods

Study design and population

This is a cross-sectional study that targeted medical students from the second year to the sixth year utilizing an online form of validated questionnaires. The study was conducted among medical students of King Abdulaziz University (KAU), Jeddah, Saudi Arabia. First-year students (preparatory year) were excluded from the study. 

The sample size was calculated using the online Raosoft calculator (Raosoft Inc., Seattle, WA, USA) for cross-sectional studies. The equation was built using a 95% confidence level, a 5% margin of error, and a 50% distribution rate. The required sample size for the current study was 382 students. A non-probability convenience sampling methodology was used to include eligible participants among medical students.

Data collection

The data collection was done through an online version of a structured questionnaire. The questionnaire included three sections. The first section inquired about the academic year and performance as measured by the grade point average (GPA). The second section included the Pittsburgh Sleep Quality Index (PSQI) (see Appendix A). The third section included the Depression, Anxiety, and Stress Scale (DASS-21) (see Appendix B). 

The questionnaire was built on Google Forms (Google LLC, Mountain View, CA, USA). The link to the electronic form was distributed to the participants using WhatsApp, the social media platform (Meta Platforms, Inc., Menlo Park, CA, USA). The data collection took place between January 2023 and February 2023.

Data analysis

The data analysis was done using SPSS Statistics version 29.0 (IBM Corp., Armonk, NY, USA). Frequency and proportions were used to interpret categorical variables. The mean and standard deviation (SD), median, and interquartile range (IQR) were used to interpret continuous variables as appropriate according to the Shapiro-Wilk normality distribution test. For correlation, the Spearman rank correlation coefficient test was used. A strong positive correlation was established when the Spearman correlation coefficient was above 0.70; moderate correlations were considered for coefficients ranging from 0.30 to 0.69; and weak correlations were considered for coefficients less than 0.30. A Kruskal-Wallis non-parametric test was used to investigate the differences in PSQI across the GPA groups. The Fisher-Freeman-Halton exact test was used to test associations between categorical variables. The significance level was set at a p-value of <0.05.

Ethical considerations

The King Abdulaziz University Faculty of Medicine's Research Ethics Committee gave its approval for the study (approval no. 550-22). Without obtaining any personal identification information, the records were safely preserved. The research purpose and nature were explained to the students to obtain informed consent in electronic form. The collected data were only used for research.

## Results

A total of 382 responses from medical students were analyzed. Sixth-year students represented the majority of the participants at 39.3%, followed by third-year students at 24.9%. The GPA of the majority of students (86.6%) was more than 3.75/5, and only 1% had less than 2.75/5. Students were asked if they had been previously diagnosed with a sleeping disorder or mental illness. While 19.1% indicated a previous diagnosis of mental illness, only 5.8% indicated a diagnosis of a sleeping disorder (Table 1). 

**Table 1:** The academic year, performance, and past history of medical diagnoses GPA: Grade point average

Questionnaire: Part 1	Groups	N	%
Year	Second-year	55	14.4%
	Third-year	95	24.9%
	Fourth-year	46	12%
	Fifth-year	36	9.4%
	Sixth-year	150	39.3%
GPA	2-2.74	4	1%
	2.75-3.74	47	12.3%
	3.75-4.49	162	42.4%
	4.50-5.00	169	44.2%
Have you been diagnosed with a sleep disorder?	Yes	22	5.8%
	No	360	94.2%
Have you been diagnosed with a mental illness?	Yes	73	19.1%
	No	309	80.9%

The PSQI was calculated for all the participants and showed a median and IQR of 9 and 6-11, respectively. The DASS-21 scale was also computed. Normal scores among participants represented the majority as follows: depression at 67%, anxiety at 63.1%, and stress at 82.2%. Depression and stress scores did not show any extremely severe cases, while anxiety showed only 0.5% as having extremely severe anxiety. The results of the DASS-21 scale are detailed in Table 2. 

**Table 2:** The prevalence and severity of mental health illnesses

Levels	Depression (%)	Anxiety (%)	Stress (%)
Normal	256 (67%)	241 (63.1%)	314 (82.2%)
Mild	53 (13.9%)	43 (11.3%)	39 (10.2%)
Moderate	59 (15.4%)	59 (15.4%)	29 (7.6%)
Severe	14 (3.7%)	37 (9.7%)	0 (0%)
Extremely severe	0 (0%)	2 (0.5%)	0 (0%)

Statistical analyses investigated the association between PSQI and the GPA of the students. The relationship was tested using Spearman’s correlation coefficient, which did not show a statistically significant association. The distribution of PSQI among the categories of GPA is shown in Table 3. 

**Table 3:** Association between sleep quality scores and academic performance of medical students *p-value calculated using the Kruskal-Wallis test PSQI: Pittsburgh Sleep Quality Index, IQR: Interquartile range, GPA: Grade point average

GPA	PSQI: Median (IQR)	Mean rank	p-value*
2-2.74	10.5 (9.5-12.5)	281.8	0.189
2.75-3.74	8 (5-11)	188.6	
3.75-4.49	8 (5-11)	182.3	
4.50-5.00	9 (6-11)	199	

The association of depression, anxiety, and stress with GPA was tested statistically. All DASS-21 components did not show a statistically significant association with GPA (Table 4). Upon further analysis, the academic year showed a weak negative correlation with PSQI, depression, and anxiety. The findings were statistically significant (Table 5). 

**Table 4:** The association between mental illnesses and different GPAs *p-value calculated using the Fisher-Freeman-Halton Exact Test GPA: Grade point average

Variables	GPAs				p-value*
	2.00 - 2.74	2.75 - 3.74	3.75 - 4.49	4.50 - 5.00	
Depression					
Normal	3 (1.2%)	30 (11.7%)	115 (44.9%)	108 (42.2%)	0.783
Mild	0 (0%)	5 (9.4%)	20 (37.7%)	28 (52.8%)	
Moderate	1 (1.7%)	10 (16.9%)	21 (35.6%)	27 (45.8%)	
Severe	0 (0%)	2 (14.3%)	6 (42.9%)	6 (42.9%)	
Extremely severe	0 (0%)	0 (0%)	0 (0%)	0 (0%)	
Anxiety					
Normal	1 (0.4%)	28 (11.6%)	107 (44.4%)	105 (43.6%)	0.433
Mild	2 (4.7%)	7 (16.3%)	16 (37.2%)	18 (41.9%)	
Moderate	1 (1.7%)	8 (13.6%)	24 (40.7%)	26 (44.1%)	
Severe	0 (0%)	4 (10.8%)	13 (35.1%)	20 (54.1%)	
Extremely severe	0 (0%)	0 (0%)	2 (100%)	0 (0%)	
Stress					
Normal	3 (1%)	38 (12.1%)	133 (42.4%)	140 (44.6%)	0.683
Mild	1 (2.6%)	5 (12.8%)	14 (35.9%)	19 (48.7%)	
Moderate	0 (0%)	4 (13.8%)	15 (51.7%)	10 (34.5%)	
Severe	0 (0%)	0 (0%)	0 (0%)	0 (0%)	
Extremely severe	0 (0%)	0 (0%)	0 (0%)	0 (0%)	

**Table 5:** Correlation between sleep quality, mental illnesses, and the academic year of the students PSQI: Pittsburgh Sleep Quality Index

Variables	Academic year	
	Spearman’s coefficient	p-value
PSQI	-0.172	<0.001
Depression	-0.183	<0.001
Anxiety	-0.165	0.001
Stress	-0.079	0.123

Further analyses showed moderately positive correlations between poor sleep quality and mental health illnesses as follows: depression (r = 0.432), anxiety (r = 0.499), and stress (r = 0.563). All the findings were statistically significant (p<0.001). 

## Discussion

Due to their heavy academic loads, medical students may experience worse sleep quality than the average member of modern society [12]. They often face numerous academic and personal challenges during their education and training, which can impact their overall academic performance. Among these challenges, sleep quality and mental health are two important factors that have been shown to significantly affect academic performance.

Our data showed that there was no significant association between the quality of sleep and the GPA of medical students. A similar study conducted in Iran investigated the effects of sleep on the academic performance of students in dental school. The findings revealed no link between sleep quantity or quality and GPA [13]. In a similar manner, a Saudi Arabian study on medical students showed no link between sleep and academic achievement. The PSQI was used as a tool to assess the sleep quality of 305 medical students. The findings revealed that academic achievement was not correlated with sleep quality [14]. However, a previous study showed a significant correlation between bad sleep quality and the academic performance of students. According to the study, medical students who reported better sleep quality had higher academic performance compared to those who reported poor sleep quality [15]. Additionally, a study found that medical students who had irregular sleep patterns had lower academic performance compared to those with regular sleep patterns. Irregular sleep patterns were defined as having a bedtime or wake-up time that varied by more than one hour on weekdays compared to weekends [16].

Research shows that individuals with better grades exhibit a statistically significant lower prevalence of depressive symptoms when compared to students with lesser grades (53.1% vs. 74.3%) [17]. In contrast, Jamil et al. found no significant association between anxiety and academic performance in medical students. The study found that although anxiety was prevalent among the participants, it did not appear to have a significant impact on their grades [18]. But mental health is a potential risk factor that can significantly affect academic performance among medical students. Research has shown that mental health problems, such as depression and anxiety, can have negative effects on academic performance [19]. According to research by Dyrbye et al., medical students who reported depressive symptoms performed worse academically than those who did not [18]. Similarly, research by Rotenstein et al. discovered that medical students who experienced burnout symptoms performed worse academically than those who did not. Burnout was described as having a diminished sense of personal success, depersonalization, and emotional weariness [19].

Our study found a significant association between academic year, sleep quality, and mental health. As the academic year progresses, the level of depression and anxiety declines, which can be explained by students adaptation to university life. However, it negatively impacts sleep quality. A study revealed that depression scores were significantly higher during the first year of medical school compared to the fourth year. The study also found that female medical students reported higher levels of depression compared to male students [19]. In a previous study, medical students reported poorer sleep quality during their clinical years compared to their pre-clinical years. The same study also found that medical students reported higher levels of sleepiness and fatigue during their clinical years [12]. Our study highlights a contradictory observation, wherein senior students showed higher sleep quality alongside an increased prevalence of mental health disorders.

It was shown that there is a slightly positive correlation between inadequate sleep and mental health issues. Many mental health conditions, such as depression, anxiety disorders, bipolar disorder, and post-traumatic stress disorder, are linked to poor sleep quality. Poor sleep quality was found to be strongly linked to a higher risk of developing depression. Also, the study discovered that people who had trouble sleeping were more likely to have severe depressive symptoms [20]. Poor sleep was found to be a significant risk factor for depression in another investigation. Sleep quality was poorer in depressed people than in healthy people [21].

Our study used validated questionnaires, which ensure that the data collected is reliable and accurate. This increases the validity of the study results and enhances confidence that the findings are trustworthy. We also recruited an adequate sample size, which increases the statistical power of the study and makes it more likely to detect significant effects or relationships. The sample is limited to medical students at King Abdulaziz University in Saudi Arabia, which may limit the generalizability of the findings. The respondents who chose to participate in the online survey may differ from those who did not, which raises the possibility of self-selection bias. For instance, individuals with strong opinions may have been more likely to participate than others, resulting in biased results. The study did not investigate gender, age, or other sociodemographic variables in its analysis. Failure to account for these variables in the analysis can increase the risk of confounding. Also, data regarding other potential confounders, such as hypothyroidism and obstructive sleep apnea, were not included in the analysis. Considering the limited number of variables, it may not be possible to explain why certain individuals or groups are more vulnerable to poor sleep quality or mental health problems. This can limit the study's ability to provide a comprehensive understanding of these issues.

## Conclusions

Our study's findings revealed inadequate sleep patterns among medical students. There was no definitive relationship between sleep quality and academic accomplishment, nor were there correlations between depression, anxiety, stress levels, and students' GPAs. Significantly, our results revealed a weak correlation between academic progression, sleep quality, and mental health. Although the severity of depression and anxiety decreased over the academic year, it negatively affected sleep. These outcomes highlight the importance of early interventions targeting mental health and sleep quality to support medical students' overall well-being and improved academic performance. We recommend conducting additional research to gain deeper insights into the mechanisms linking sleep and mental health in medical students and to identify therapeutic strategies for mental and sleep disorders.

## Appendices

Appendix A

The Pittsburgh Sleep Quality Index (PSQI) 

Participants were briefed that the PSQI questionnaire (Table 6) would assess their usual sleep habits during the past month only [22]. They were asked to provide answers that indicate the most accurate reply for the majority of days and nights in the past month. And they were requested to answer all the questions.

**Table 6:** Items of the PSQI PSQI: Pittsburgh Sleep Quality Index

Questions	Answer			
1. During the past month, what time have you usually gone to bed at night?				
2. During the past month, how long (in minutes) has it usually taken you to fall asleep each night?				
3. During the past month, what time have you usually gotten up in the morning?				
4. During the past month, how many hours of actual sleep did you get at night?				
5. During the past month, how often have you had trouble sleeping because you...	Not during the past = 0	Less than once a week = 1	Once or twice a week = 2	Three or more times a week = 3
a. Cannot get to sleep within 30 minutes				
b. Wake up in the middle of the night or early morning				
c. Have to get up to use the bathroom				
d. Cannot breathe comfortably				
e. Cough or snore loudly				
f. Feel too cold				
g. Feel too hot				
h. Have bad dreams				
i. Have pain				
j. Other reason(s), please describe (scoring based on description)				
6. During the past month, how often have you taken medicine to help you sleep?				
7. During the past month, how often have you had trouble staying awake while driving, eating meals, or engaging in social activity?				
8. During the past month, how much of a problem has it been for you to keep up enough enthusiasm to get things done?	No problem at all	Only a very slight problem	Somewhat of a problem	A very big problem
9. During the past month, how would you rate your sleep quality overall?	Very good = 0	Fairly good = 1	Fairly bad = 2	Very bad = 3

The scoring system for the PSQI is shown in Table 7.

**Table 7:** Scoring system for the PSQI *Sleep efficiency = (# hours slept / # hours in bed) X 100% Hours slept: Determined from question 4; Hours in bed: Calculated from responses to questions 1 and 3 PSQI: Pittsburgh Sleep Quality Index

Component		Responses	Score
Subjective sleep quality	Q9	Very good	0
		Fairly good	1
		Fairly bad	2
		Very bad	3
Component 1 score			
Sleep latency	Q2	< 15 minutes	0
		16-30 minutes	1
		31-60 minutes	2
		> 60 minutes	3
	Q5a	Not during the past month	0
		Less than once a week	1
		Once or twice a week	2
		Three or more times a week	3
	Q2 and Q5a subscores	0	0
		1-2	1
		3-4	2
		5-6	3
Component 2 score			
Sleep duration	Q4	> 7 hours	0
		6-7 hours	1
		5-6 hours	2
		< 5 hours	3
Component 3 score			
Sleep efficiency*	Q 1, 3, and 4	> 85%	0
		75-84%	1
		65-74%	2
		< 65%	3
Component 4 score			
Sleep disturbance	Questions 5b to 5j should be scored as follows	Not during the past month	0
		Less than once a week	1
		Once or twice a week	2
		Three or more times a week	3
	Sum of 5b to 5j scores	0	0
		1-9	1
		19-18	2
		19-27	3
Component 5 score			
Use of sleep medication	Q6	Not during the past month	0
		Less than once a week	1
		Once or twice a week	2
		Three or more times a week	3
Component 6 score			
Daytime dysfunction	Q7	Not during the past month	0
		Less than once a week	1
		Once or twice a week	2
		Three or more times a week	3
	Q8	No problem at all	0
		Only a very slight problem	1
		Somewhat of a problem	2
		A very big problem	3
	Sum of Q7 and Q8 subscores	0	0
		1-2	1
		3-4	2
		5-6	3
Component 7 score			
Global PSQI Score: Sum of seven component scores			

Appendix B

The Depression, Anxiety, and Stress Scale (DASS-21) Questionnaire

Participants were asked to read each statement of the DASS-21 questionnaire (Table 8) and circle a number, i.e., 0, 1, 2, or 3 which indicates how much the statement applied to them over the past week [23]. They were assured that there were no right or wrong answers and requested not to spend too much time on any statement.

**Table 8:** Items of the DASS-21 questionnaire Rating scale: 0 = Did not apply to me at all; 1 = Applied to me to some degree, or some of the time; 2 = Applied to me to a considerable degree or a good part of the time; 3 = Applied to me very much or most of the time DASS: Depression, Anxiety, and Stress Scale

Statement	0	1	2	3
1 (s) I found it hard to wind down				
2 (a) I was aware of dryness of my mouth				
3 (d) I couldn’t seem to experience any positive feeling at all				
4 (a) I experienced breathing difficulty (e.g., rapid breathing)				
5 (d) I found it difficult to work up the initiative to do things				
6 (s) I tended to over-react to situations				
7 (a) I experienced trembling (e.g., in the hands)				
8 (s) I felt that I was using a lot of nervous energy				
9 (a) I was worried about situations where I might panic				
10 (d) I felt that I had nothing to look forward to				
11 (s) I found myself getting agitated				
12 (s) I found it difficult to relax				
13 (d) I felt down-hearted and blue				
14 (s) I was intolerant of anything that kept me from doing things				
15 (a) I felt I was close to panic				
16 (d) I was unable to become enthusiastic about anything				
17 (d) I felt I wasn’t worth much as a person				
18 (s) I felt that I was rather touchy				
19 (a) I was aware of the action of my heart in the absence of exertion				
20 (a) I felt scared without any good reason				
21 (d) I felt that life was meaningless				

The scoring system for the DASS-21 is shown in Table 9. Each scale contains 7 items, marked by letters after the numbering of each statement. After the calculation of each scale, the results should be interpreted. Scores on the DASS-21 will need to be multiplied by 2 to calculate the final score.

**Table 9:** Recommended cut-off scores for conventional severity labels (normal, moderate, severe)

Levels	Depression	Anxiety	Stress
Normal	0-9	0-7	0-14
Mild	10-13	8-9	15-18
Moderate	14-20	10-14	19-25
Severe	21-27	15-19	26-33
Extremely Severe	28+	20+	34+
